# Inflammation changes the expression of neuropeptide Y receptors in the pig myometrium and their role in the uterine contractility

**DOI:** 10.1371/journal.pone.0236044

**Published:** 2020-07-10

**Authors:** Barbara Jana, Jarosław Całka, Katarzyna Palus

**Affiliations:** 1 Division of Reproductive Biology, Institute of Animal Reproduction and Food Research of the Polish Academy of Sciences, Olsztyn, Poland; 2 Division of Clinical Physiology, Faculty of Veterinary Medicine, University of Warmia and Mazury, Olsztyn, Poland; Medical University Innsbruck, AUSTRIA

## Abstract

In the experiment the influence of inflammation on neuropeptide Y (NPY) receptor subtype 1 (Y1Rs) and 2 (Y2Rs) expression pattern in the gilt myometrium and NPY effect alone or with Y1Rs and Y2Rs antagonists on contractility of an inflamed uterus were recognized. On Day 3 of the estrous cycle, either *E*.*coli* suspension (*E*.*coli* group) or saline (SAL group) were administered into uterine horns. In the control gilts (CON group), only laparotomy was carried out. Eight days later, *E*.*coli* treatment evoked severe acute endometritis, significantly reduced Y1Rs mRNA and protein expression and increased Y2Rs protein expression in myometrium in relation to the CON and SAL groups. Compared to period before NPY application, NPY (10^−7^ M) significantly reduced amplitude in myometrium and endometrium/myometrium and frequency in myometrium of the CON and SAL groups and amplitude in endometrium/myometrium and frequency in strips of the *E*.*coli* group. In this group, after using NPY (10^−7^ M), the amplitude rose significantly in both kinds of strips, while frequency fell in endometrium/myometrium in relation to the SAL group. In the CON and SAL groups, NPY (10^−7^ M) with Y1Rs antagonist significantly increased the amplitude in endometrium/myometrium, while with Y2Rs antagonist caused a rise in myometrium. In the *E*.*coli* group after using Y1Rs or Y2Rs antagonist and NPY (10^−7^ M) amplitude did not significantly change in endometrium/myometrium, and this parameter was significantly lower after using the NPY and Y1Rs antagonist than in other groups. Y1Rs antagonist and NPY (10^−8^, 10^−7^ M) significantly increased frequency in endometrium/myometrium of the CON and SAL groups. In the *E*.*coli* group, Y1Rs or Y2Rs antagonists and NPY (10^−7^ M) significantly increased frequency in myometrium and it was significantly higher than in the CON group. Inflammation changes Y1Rs and Y2Rs expression in myometrium of the inflamed pig uterus and NPY reduces this organ contractility by Y1Rs and Y2Rs.

## Introduction

Uterine inflammatory process is a very common condition in domestic animals which leads to economic losses [1, 2]. This disorder is generally evoked by bacteria and found predominantly after parturition as a result of impaired uterine involution and/or immunological response. States such as difficult labor, fetal membrane retention and uterine contractility disturbances are conductive to the beginning of uterine inflammation [3]. In more advanced cases of inflammatory process, in the uterine cavity inflammatory exudate (mucopurulent) is present. Many therapies are used for endometritis/metritis. However, positive effects of uterine inflammation treatment do not apply to all animals. It may be related to a weaker ability of the myometrium (MYO) to contract or with loses of this activity [4]. When synthetized and released in great amounts prostaglandin (PG)F_2_α, PGE_2_ [5], PGI_2_ [6], leukotrienes (LT)B_4_ and LTC_4_ [7] in porcine uterus with inflammation participate in the contractile activity of this organ [6, 8, 9, 10].

Neuropeptide Y (NPY) belongs, along with peptide YY (PYY), to the pancreatic polypeptide (PP) family [11]. The presence of NPY was revealed in pig uterus-innervating noradrenergic neurons [12] and rat [13] inferior mesenteric ganglion and in cholinergic and noradrenergic neurons in the pig paracervical ganglion [14]. In the uterus of humans [15], pigs [16] and rats [17] NPY-immunoreactive nerve fibers are present in the vicinity of blood vessels, endometrial glands and myocytes of the MYO. The peptides of the PP family act by six subtypes of G protein-coupled Y-family receptors (YRs), from Y1 to Y6. NPY and PYY preferentially bind Y1Rs, Y2Rs and Y5Rs, while PP mainly acts via Y4Rs [11]. It was reported that under physiological conditions NPY in a dose-dependent manner stimulates the spontaneous contractility in the rabbit uterine body [18] and increases the contractility of rat MYO through Y1Rs [19]. This peptide did not affect the contractile activity of the rat MYO, but inhibited contractility mediated by cholinergic nerve fibers in the uterine cervix [20]. Similarly, a direct contractile effect of NPY was not demonstrated in the human MYO or oviduct [21, 22]. NPY induces the contraction of guinea pig uterine arteries [23, 24] acting predominantly via Y1Rs [25]. In the uterine artery, NPY inhibits relaxation produced by vasoactive intestinal peptide (VIP) in guinea pigs [23] and potentiates the noradrenergic contractile response in women [26]. Data also exist showing the nerve fibers expressing NPY in the pseudo-capsule of uterine fibroids in women, suggesting its effect on the contractility of muscles and their healing [27]. NPY-immunoreactive nerve fibers and increased expression of Y1Rs were revealed in the uterosacral ligament in patients with pelvic organ prolapse, indicating a role of these factors in local flow and structural changes of pelvic supporting tissue [28].

In addition, NPY is a potent modulator of the immune responses during inflammation, for example, by regulating neutrophil chemotaxis, nitric oxide production and T helper cell differentiation [29]. NPY and its receptors (Y1Rs, Y2Rs) are involved in inflammatory processes of intestines [30], chronic pain [31] as well as in cardiovascular disease pathogenesis [11]. To date, only the contribution of main autonomic neurotransmitters–noradrenaline (NA) and acetylcholine (ACh) in the contractility of the inflamed uterus [6, 9, 32] and the expression of noradrenergic receptors in pathologically-changed organ [33] were evaluated. In view of these data, it is hypothesized that uterine inflammation changes the expression of YRs and that it conditions the NPY effect on the contractility of an inflamed uterus. Understanding the receptor background of the NPY effect may be significant to the course and/or outcome of uterine inflammation. Thus, the aim of this study was to recognize the influence of inflammation on Y1Rs and Y2Rs expression in gilt MYO and their functional relevance in contractility of the uterus.

## Materials and methods

### Experimental animals

The experiment was performed in accordance with the principles of animal care (National Institute of Health publication No. 86–23, revised in 1985) and the specifics of the national law concerning animal protection. The Local Ethics Committee of the University of Warmia and Mazury in Olsztyn approved all procedures and granted consent (no. 65/2015).

This study was carried out on 15 gilts (crossbred Large White x Landrace) at the age of 7–8 months and body weight (BW) of approximately 90–120 kg. By using a tester boar, behavioral estrus was detected. In all examined animals, disruption in reproductive state did not occur: vaginal discharges were not revealed and the second estrous cycle was regular. Three days before the start of the research, the gilts were transported from a farm to the local animal house in the Faculty of Veterinary Medicine, University of Warmia and Mazury, Olsztyn (Poland). They were kept in normal laboratory conditions. The gilts were maintained in individual pens with an area of about 5 m^2^ under the following conditions: natural daylight -14.5±1.5 h, night—9.5±1.5 h and temperature 18±2°C. They were fed with commercial diets according to their nutritional requirements and had access to water. During the whole study clinical examination of all gilts was carried out (a least 3 times a day) by a veterinarian. Special attention was paid to changes in animal behavior related to pain. No deviations in the overall animal health state were found. The gilts were not medically treated.

### Experimental procedure

The study procedure has been reported earlier [6]. On day 3 of the second estrous cycle (day 0 of the study), fifteen gilts were divided (randomly) into three groups: the *E*. *coli*- (*E*. *coli* group, n = 5) and saline (SAL group, n = 5) -treated gilts, and the control (CON group, n = 5)–animals subjected to “sham” operation (details are below).

All gilts were pre-medicated with atropine (0.05 mg/kg BW; administered intramuscularly /i.m/, Atropinum sulf. WZF, Warszawskie Zakłady Farmaceutyczne Polfa S.A., Warsaw, Poland), azaperone (2 mg/kg BW; administered i.m., Stresnil, Janssen Pharmaceutica, Beerse, Belgium) and ketamine hydrochloride (10 mg/kg BW; administered intravenously /i.v/, Ketamina, Biowet, Puławy, Poland). General anesthesia was reached with ketamine hydrochloride and prolonged by supplementary doses of this medicine (1 mg/kg BW every 5 min, administered i.v.). After laparotomy was done, either 50 ml of *E*. *coli* suspension (*E*. *coli* group; 1 ml of suspension containing 10^9^ colony-forming units, strain O25:K23/a/:H1; National Veterinary Research Institute, Department of Microbiology, Puławy, Poland), or 50 ml of saline solution (SAL group) were administered into both uterine horns. By the use of a syringe with a 0.8 mm gauge needle, bacterial suspension or saline were injected into each uterine horn in 5 places (10 ml of solution per each injection, a similar distance between the places of the injections was kept). So that the solution does not flow from the uterus, the injection places were pressed gently with gauze, and then wiped with gauze and saline. Next, the horns were massaged to evenly distribute *E*. *coli* suspension or saline. In the animals of the CON group, only laparotomy was carried out. After 8 days (the expected day 11 of the estrous cycle), euthanasia of animals was performed by the use of an overdose of ketamine hydrochloride (administered i.v.) and the uteri were collected. Horns of uteri were cut open along their long axis and macroscopic assessment of the endometrial layer was carried out. Attention was paid to the presence of inflammatory exudate in the uterus (its quantity and nature) and the appearance of the endometrial layer: color, blood vessels visibility, presence and intensity of edema. Next, fragments of the uterine horn were obtained (from the paraoviductal, middle, paracervical parts of the uteri) for real-time reverse transcriptase-polymerase chain reaction (real-time RT-PCR) and Western blot analyses. For this purpose, endometrial and myometrial layers were separated using a scalpel blade. The distribution of these layers was estimated by the use of a dissecting microscope. The myometrial pieces of 1 cm long (thickness of entire layer) were immediately shock-frozen in liquid nitrogen and stored at -80°C for real-time RT and Western blot studies. The collection of uterine horn fragments, separation of both uterine layers and their freezing were carried out under sterile conditions to avoid contamination. Pieces of the uterine horns were collected from three parts of the organ for histological and immunofluorescent studies. First, they were divided into smaller pieces and fixed in 4% paraformaldehyde solution (pH 7.4) After 24 h, pieces were washed in 0.1 M phosphate buffered-saline (PBS, pH 7.4). To perform immunofluorescent staining, tissues were cryoprotected in 18% sucrose until sectioning, while for the histopathological examination they were routinely embedded in paraffin. The findings of macroscopic and histological assessment of uteri were presented previously [33]. The uterine horns fragments from the middle part, were placed on ice and transported to the laboratory within 5 min after collection, for study of the contractility.

### RNA extraction, and real-time RT-PCR

Total RNA was isolated from myometrial tissues. They were homogenized in TRI Reagent solution (Invitrogen, Thermo Fisher Scientific, USA) using a FastPrep 24 homogenizer (MP Biomedicals, LCC, USA). For phase separation, a BCP reagent (Molecular Research Center Inc., USA) was used, and the RNA was then purified by using an RNeasy Mini Kit (QIAGEN, USA), in accordance with the manufacturer’s instructions. RNA was stored until further use at -80°C in RNase-free water with the addition of RNAse Inhibitor (Applied Biosystems, Thermo Fisher Scientific, USA). The quality and quantity of extracted RNA was determined by the use of NanoDrop 1000 (Thermo Fisher Scientific, USA) and Agilent 2100 Bioanalyzer (Agilent Technologies, USA). RNA with an RNA Integrity number ranging 7.0–9.6 was used in real-time RT-PCR.

Real-time RT-PCR was carried out by the use of TaqMan tests (Table 1) and a one-step PCR Master mix (Applied Biosystems). Each reaction (10μl) contained: 15 ng of total RNA in a volume of 3 μl, 5 μl 2 x TaqMan RT-PCR Mix, 0.25 μl 40 x TaqMan RT Enzyme Mix, 0.5 μl 20 x TaqMan Gene Expression Assays and 1.25 μl RNase-free water (Applied Biosystems). The real-time RT-PCR reaction was performed in duplicates in 384-well plates using the following conditions: reverse transcription for 15 min at 48°C, initial denaturation for 10 min at 95°C, followed by 45 cycles of 15 s of denaturation at 95°C and then 1 min of annealing at 60°C, in an ABI Prism 7900HT system (Applied Biosystems). The negative control was prepared by replacing the RNA template with RNase free water. Data obtained were analyzed by the use of the Miner method [34]. The NormFinder algorithm was utilized to choose the most stable housekeeping gene among: β-actin (ACTB), hypoxanthine guanine phosphoribosyl transferase (HPRT), glyceraldehyde-3-phosphate dehydrogenase (GAPDH) [35]. The best stability value was determined for the combination of ACTB and GAPDH genes (0.171). The expression levels for each target gene were normalized relatively to the geometric mean of ACTB and GAPDH gene expression.

**Table 1:** TaqMann assays used in this study.

Symbol	Name	Assay No.
*NPY1R*	*neuropeptyde Y receptor Y1*	*Ss03394613_g1*
*NPY2R*	*neuropeptyde Y receptor Y2*	*Ss03394187_s1*
*GAPDH*	*glyceraldehyde-3-phosphate dehydrogenase*	*Ss03375435_u1*
*ACTB*	*β-actin*	*Ss03376081_u1*
*HPRT*	*hypoxanthine guanine phosphoribosyl transferase*	*Ss03388274_m1*

### Western blotting

The expression of Y1Rs and Y2Rs proteins in the myometrial tissues was estimated as published previously [36]. Pieces of the MYO were homogenized on ice with a cold buffer (50 mmol/l Tris-HCl, pH 8.0; 150 mmol/l NaCl; 1% Triton X-100, 10 mg/ml aprotinin, 52 mmol/l leupeptin, 1 mmol/l pepstatin A, 1 mmol/l EDTA, 1 mol/l PMSF) and centrifuged (10 min; 2,500g at 4°C). The supernatants were centrifuged (1 h; 17,500g at 4°C) and the obtained supernatants were then stored at -80°C. The protein content was determined by Bradford method [37]. The myometrial portions (20 μg) were dissolved in a sodium dodecyl sulfate (SDS) gel-loading buffer (50 mmol/l Tris-HCl, pH 6.8; 4% SDS, 20% glycerol and 2% β-mercaptoethanol), heated (95°C, 4 min) and separated by 10% SDS-polyacrylamide gel electrophoresis. The separated proteins were electro-blotted onto 0.22 μm nitrocellulose membrane in transfer buffer (20 mmol/l Tris-HCl buffer, pH 8.2; 150 mmol/l glycine, 20% methanol, 0.05% SDS). The nonspecific binding sites were blocked by incubation with 5% fat-free dry milk in a TBS-T buffer at room temperature (RT) for 1.5 h. The nitrocellulose membranes were incubated overnight (at 4°C) with primary polyclonal antibodies including: rabbit anti-human Y1Rs antibody (diluted 1:500, cat. no. AP01221PU-N) and rabbit anti-human Y2Rs antibody (diluted 1:1000, cat. no. TA314282) both from Acris an OriGene Company. After being washed in TBS-T buffer, the nitrocellulose membranes were incubated with biotinylated goat anti-rabbit IgG (diluted 1:3000, cat. no. PK-6101, Vectastain Elite ABC-HRP Kit, Vector Labs, Burlingame, CA, USA) for 1 h, at RT. Visualization of antibody binding was carried out by incubation with a freshly prepared mixture of 3, 30-diaminobenzidine tetrahydrochloride (DAB, cat. no. D5637) and H_2_O_2_ in Tris-buffered saline (pH 7.2), for 3–4 min. Images were captured and quantitated by the use of a Quantity-One system (VersaDoc 4000M imaging system, Bio-Rad Laboratories, Hercules, CA, USA). The band density was normalized based on GAPDH protein expression.

### Immunofluorescence

To determine the Y1Rs and Y2Rs distribution, fragments of uterine horns were cut in cryostat (Reichert-Jung, Nußloch, Germany). Sections (10-μm-thick) were subjected to the single-immunofluorescence method [38]. The sections were dried (RT, 30 min), washed in 0.1 M PBS (pH 7.4; 3 x 15 min) and incubated in blocking buffer, containing 0.1 M PBS, 10% normal goat serum (MP Biomedicals, Solon, OH, USA), 0.1% bovine serum albumin (Sigma-Aldrich, St. Louis, MO, USA), 0.05% Thimerosal (Sigma-Aldrich, St. Louis, MO, USA), 1% Triton X-100 (Sigma-Aldrich, St. Louis, MO, USA) and 0.01% NaN3 at RT for 1 h. After further rinsing in PBS (3 x 15 min), tissues were incubated overnight (RT) in a humidity chamber, with primary antibodies, the same as for Western blot analysis against Y1Rs and Y2Rs, both diluted 1:100. The next day, following washing in PBS (3 x 15 min), the sections were incubated with biotinylated anti-rabbit IgG (diluted 1:1000, cat. no AP132B, Chemicon International, Temecula, CA, USA) for 1 h, at RT and subsequently with carbocyanine 3 (CY3)-conjugated streptavidin (diluted 1:9000, cat. no. 016160084, Jackson ImmunoResearch Labs, West Grove, PA, USA) for 1 h at RT. Negative control staining was accomplished by replacing the primary antibodies with the same concentration of rabbit normal IgG. The kind of myometrial cells displaying immunoreactivity for studied receptors was estimated using an Olympus BX51 microscope (Olympus Consilio sp. z.o.o., Warsaw, Poland) equipped with epi-fluorescence and appropriate filter sets.

### Isolation of the uterine strips and recordings of their isometric contractility

**Fig 1:**
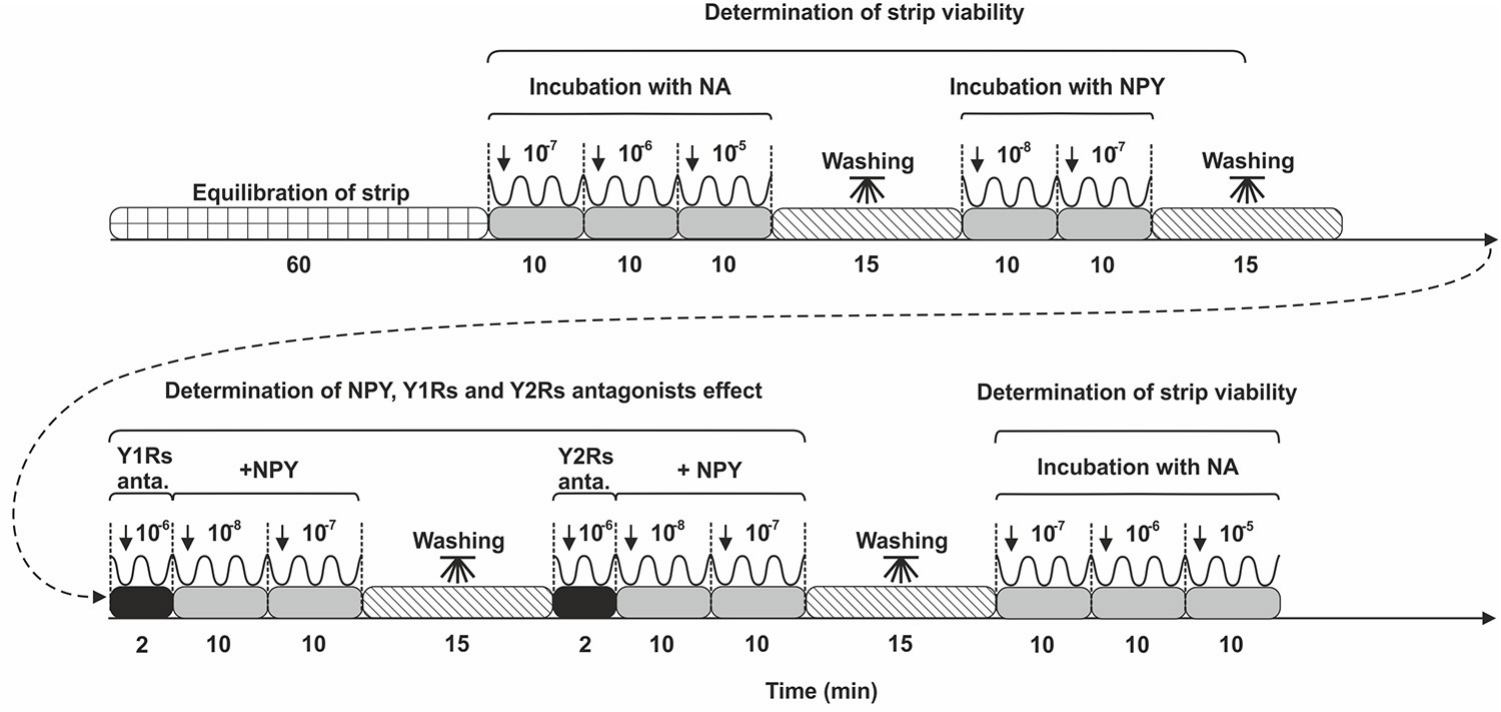
Treatment of the uterine strips. Scheme presenting sequence of treatment of uterine strips. NA—noradrenaline; NPY–neuropeptide Y; Y1Rs anta., NPY receptor subtype 1 antagonist; Y2Rs anta., NPY receptor subtype 2 antagonist. Concentrations of the used substances are given in moles.

Following strips (size 3 x 5 mm) were prepared from the horns of uterus: MYO and endometrium (ENDO)/MYO [9]. In brief, the strips were rinsed in saline and mounted between two stainless steel hooks in a 10-ml organ bath (Radnoti Unit Tissue Organ Bath System type 159920, Germany) at 5 mN rest tension. The strips were kept in a Krebs-Ringer solution (mM/l: NaCl, 120.3; KCl, 5.9; CaCl_2_, 2.5; MCl_2_, 1.2; NaHCO_3_, 15.5; glucose, 11.5; pH 7.4). The solution was continuously oxygenated (95% O_2_ and 5% CO_2_) at 37°C. The treatment scheme of uterine strips is presents on Fig 1. Following equilibration, the contractility of the strips was measured for 60 min. Contractile parameters such as tension, amplitude and frequency were measured by the use of a force displacement transducer, and recorded and analyzed on a computer with PowerChart software (Chart v5, scope v5, AD Instruments). First, the strips were treated with NA (Levonor, Warszawskie Zakłady Farmaceutyczne Polfa, Poland) at increasing concentrations (10^−7^, 10^−6^, 10^−5^ M) to estimate the viability of strips and their suitability for further experiment stages. The findings on the effect of NA have been reported previously [32]. Afterwards, the stripes were treated by NPY at doses 10^−8^ and 10^−7^ M (cat. no. H-4430.0500, Bachem). The influence of each dose was determined for 10 min. The contractility of uterine strips was recorded also after adding NPY in the presence of Y1Rs and Y2Rs antagonists (BIBO 3304 trifluoroacetate and CYM 9484, cat. no. 2412 and 4606, respectively; Tocris Biotechnology, USA). First, stripes were incubated for 2 min with the Y1Rs and Y2Rs antagonists (a dose: 10^−6^ M) and then NPY (doses: 10^−8^, 10^−7^ M) was applied and the measurements were recorded for 10 min. Following each measurement, the stripes were washed (three times) in 15 ml of phosphate buffer. NA was again added at the doses given above to re-evaluate the uterine strips viability. Only data for which the difference in NA effect at the start and end of the research was less than 20% were included in this experiment. NA concentrations were selected based on earlier studies [8, 9].

### Statistical analyses

The contractile activity of uterus was estimated based on following parameters: tension (resting/baseline tension expressed in mN), amplitude (the difference between minimum and maximum value for a single contraction expressed in mN), frequency (the number of peaks). Mean (±SEM) value of each parameter calculated for particular group before the application of the substances (pre-treatment 10-min. period) was accepted as 100% (basal value). Mean (±SEM) values of the parameters for particular group after substances addition (NA at each dose 10^−7^, 10^−6^ and 10^−5^ M; NPY at each dose 10^−8^ and 10^−7^ M; NPY at each dose 10^−8^ and 10^−7^ M with Y1Rs and Y2Rs antagonists at a dose of 10^−6^ M) were also calculated for 10-min periods. The effects of particular substances at each dose were expressed as a percentage (mean±SEM) in relation to the basal (pre-treatment period) tension, amplitude and frequency. In the contractile study, the Bonferroni test was used to determine the statistical significance between 1) mean values before (basal values) and after each treatment in each group, and 2) mean values between groups under the same treatment (ANOVA, InStat Graph Pad, San Diego, CA). Mean (±SEM) levels of Y1Rs and Y2Rs mRNA and protein expression were calculated for each group and the data were statistically assessed by the Bonferroni test. Differences at P<0.05 were considered statistically significant. Before the study, a statistical power calculation was not performed. The number of gilts in the examined group was based on the earlier experiments, in which 5 animals were used for uterine studies.

## Results

### Messenger RNA expression of Y1Rs and Y2Rs

**Fig 2:**
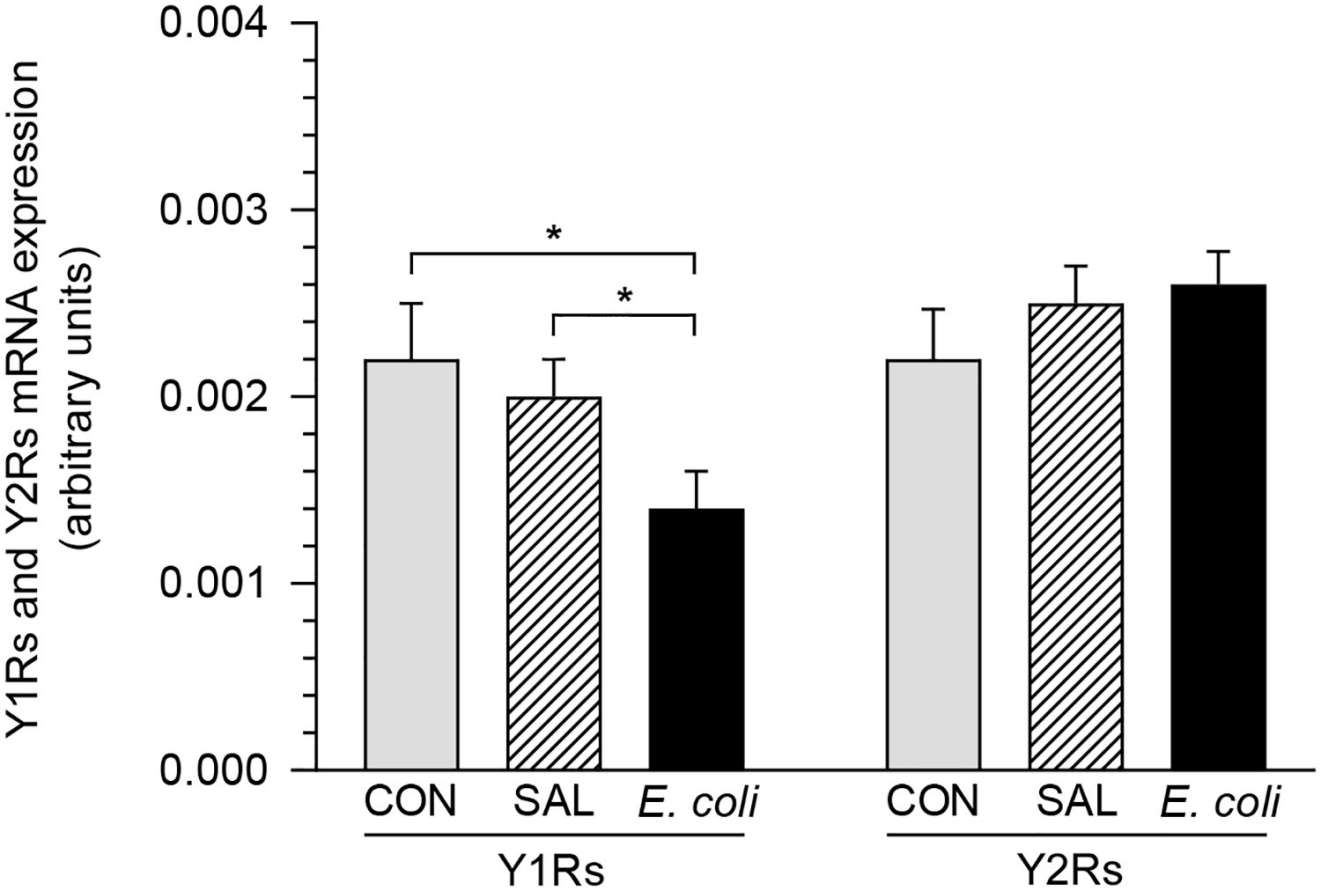
Messenger RNA levels of Y1Rs and Y2Rs. Messenger RNA levels of Y1Rs and Y2Rs in the myometrium of gilts from the control (CON), saline (SAL) and *E*. *coli* (*E*. *coli*) groups (n = 5 for each group). Results of quantification by real-time PCR are expressed as the mean±SEM of ratios relative to glyceraldehyde-3-phosphate dehydrogenase (GAPDH). *P<0.05—indicates differences between groups for the same receptor.

After intrauterine *E*. *coli* injection, the Y1Rs mRNA expression in the MYO was lower (P<0.05) than in the CON and SAL groups (Fig 2). The level of Y2Rs mRNA expression in the MYO of the control, saline- and *E*. *coli*-injected uteri did not differ significantly.

### Protein expression of Y1Rs and Y2Rs

**Fig 3:**
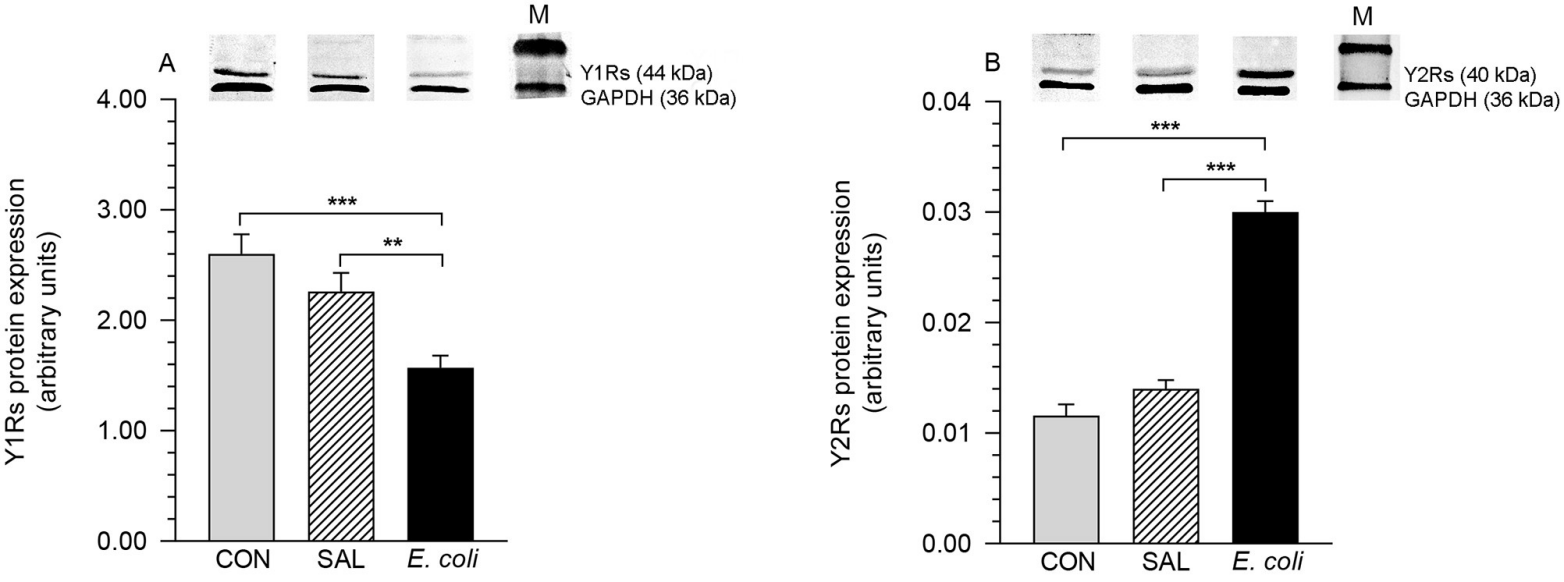
Western blotting of Y1Rs and Y2Rs. Western blotting of Y1Rs (A) and Y2Rs (B) in the myometrium of gilts from the control (CON), saline (SAL) and *E*. *coli* (*E*. *coli*) groups (n = 5 for each group). Upper panels show representative blots. Results of quantification of protein levels are expressed as the mean±SEM of ratios relative to glyceraldehyde-3-phosphate dehydrogenase (GAPDH). **P<0.01, ***P<0.001—indicate differences between groups for the same receptor. M, marker.

Protein bands corresponded to the molecular weight of Y1Rs and Y2Rs—44 and 40 kDa (Fig 3A and 3B, respectively). In the MYO following *E*. *coli* injection, the level of Y1Rs protein expression was lower than in the control (P<0.001) and saline-injected (P<0.01) uteri (Fig 3A). The level of Y2Rs protein in myometrial samples from and *E*. *coli*-injected uteri was enhanced (P<0.001) compared to the control and the saline-treated uteri (Fig 3B).

### Localization of Y1Rs and Y2Rs

**Fig 4:**
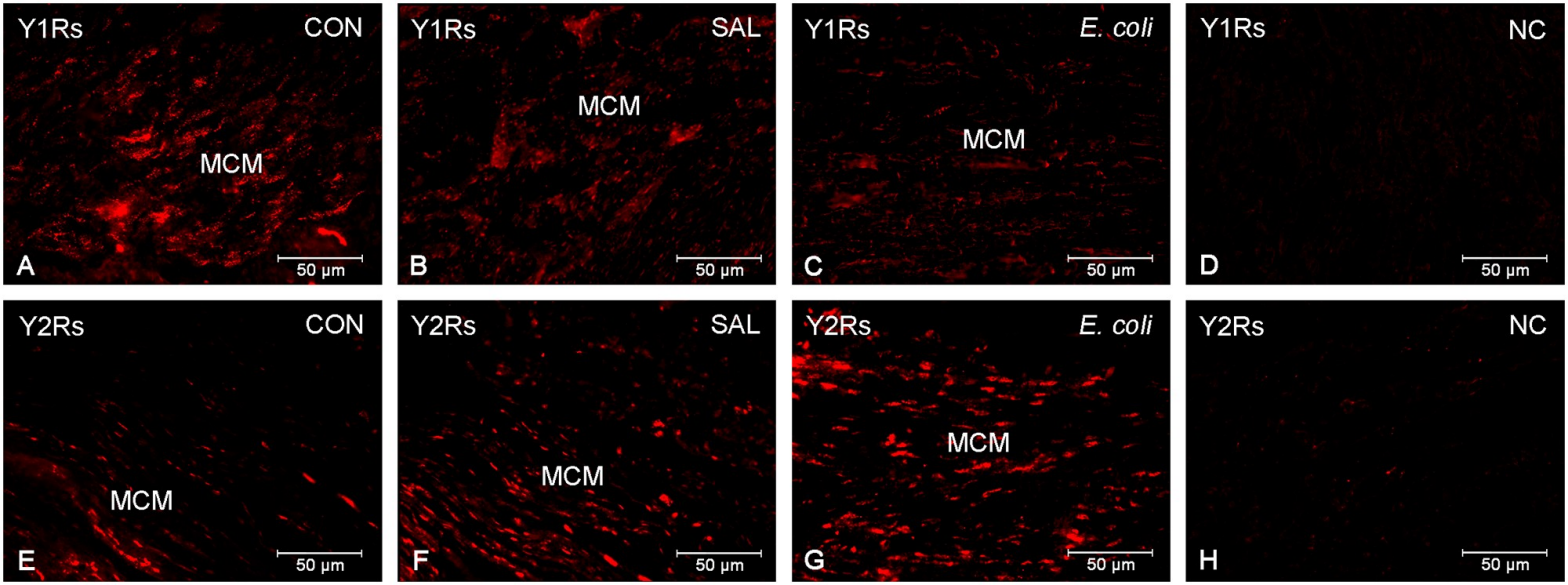
Localization of Y1Rs and Y2Rs. Representative images of Y1Rs (A-C) and Y2Rs (E-G) in the myometrium of gilts from the control (CON), saline (SAL) and *E*. *coli* (*E*. *coli*) groups. Positive reaction to Y1Rs is visible in myometrial muscular cells (MCM) of the control (A), saline- (B) and bacteria (C)-injected uteri. Expression of Y2Rs display MCM in the control (E), saline-injected (F) and inflamed (G) uteri. Negative control (NC) for Y1Rs (D) and Y2Rs (H) after replacing primary antibodies with rabbit normal IgG.

No labelling of Y1Rs (Fig 4D) and Y2Rs (Fig 4H) was detected when the primary antibodies were replaced by normal rabbit IgG. Immunofluoroscence revealed the presence of Y1Rs and Y2Rs in the muscle cells and blood vessels (endothelium, muscle layer) of the circular and longitudinal layers of the MYO of gilts from the CON (Fig 4A and 4E), SAL (Fig 4B and 4F) and *E*. *coli* (Fig 4C and 4G) groups.

### Effect of NPY on the contractile activity

#### Myometrium

**Fig 5:**
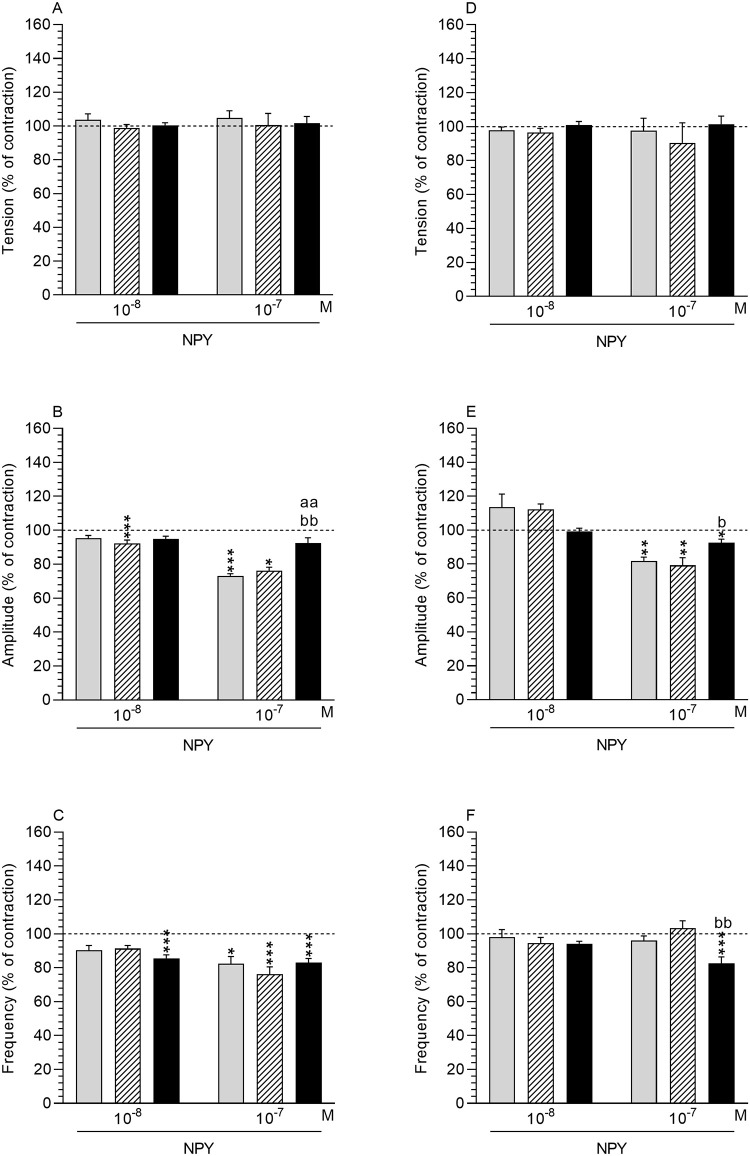
Effect of neuropeptide Y (NPY) on the contractile activity. Effect of neuropeptide Y (NPY) on the tension (A, B), amplitude (C, D) and frequency (E, F) of contraction in myometrium (A-C) and endometrium/myometrium (D-F) strips from the control (grey bars), saline- (hatched bars) and *E*. *coli* (black bars) -treated uteri of gilts. Data obtained from five experiments (gilts, in each group). Effects of particular doses of NPY are presented as a percentage (mean±SEM) in relation to the basal (pre-treatment period) tension, amplitude and frequency, accepted as 100% (horizontal lines). *P<0.05, **P<0.01, ***P<0.001—indicate differences in comparison to the basal values in each group; ^aa^P<0.01—indicates difference between the control and *E*. *coli*-treated groups for the same treatment; ^b^P<0.05, ^bb^P<0.01- indicate differences between the saline- and *E*. *coli*-treated groups for the same treatment.

*NPY effect in comparison to the period before NPY administration*. *Tension*: In the control, saline- and *E*. *coli*-injected gilts, the tension in the MYO after the use of NPY (10^−8^, 10^−7^ M) did not significantly change (Fig 5A).

*Amplitude*: NPY at doses of 10^−8^ (P<0.001) and 10^−7^ (P<0.05) M reduced amplitude in the MYO of the saline-treated uteri and at a dose of 10^−7^ M in the control uteri (P<0.001) (Fig 5B).

*Frequency*: In the MYO of the control (P<0.05) and saline-injected (P<0.001) uteri, NPY at a dose of 10^−7^ M decreased the frequency (Fig 5C). Similar results (P<0.001) were found in the MYO of *E*. *coli*-treated uteri after the use of NPY at doses of 10^−8^ and 10^−7^ M.

*Comparison of NPY effect between groups*. *Tension and frequency*: In all examined groups, the tension and frequency in the MYO were similar after the application of NPY (10^−8^, 10^−7^ M) (Fig 5A and 5C).

*Amplitude*: Compared to the control and saline-injected uteri, the amplitude in the MYO of the inflamed uteri was higher (P<0.01) in response to NPY at a dose of 10^−7^ M (Fig 5B).

#### Endometrium/myometrium

*NPY effect in comparison to the period before NPY administration*. *Tension*: In the control, saline- and *E*. *coli*-treated gilts, NPY (10^−8^, 10^−7^ M) did not significantly change the tension in ENDO/MYO (Fig 5D).

*Amplitude*: The drop in the amplitude in ENDO/MYO of the control, saline- (P<0.01) and *E*. *coli* (P<0.05) -treated gilts was revealed in response to NPY at a dose of 10^−7^ M (Fig 5E). *Frequency*: NPY at a dose of 10^−7^ M reduced (P<0.001) the frequency in ENDO/MYO of the *E*. *coli-*treated uteri (Fig 5F).

*Comparison of NPY effect between groups*. *Tension*: The tension in ENDO/MYO of the control, saline- and *E*. *coli*-treated gilts did not differ significantly between the control, saline- and *E*. *coli*-treated gilts after using NPY (10^−8^, 10^−7^ M) (Fig 5D).

*Amplitude*: After the addition NPY at a dose of 10^−7^ M the amplitude in ENDO/MYO of the inflamed uteri was higher (P<0.05) than in the saline-treated uteri (Fig 5E).

*Frequency*: In the ENDO/MYO after bacteria administration, the frequency of contraction was lower (P<0.01) compared to the saline-treated uteri (Fig 5F).

### Effect of NPY in the presence of Y1Rs antagonist

#### Myometrium

**Fig 6:**
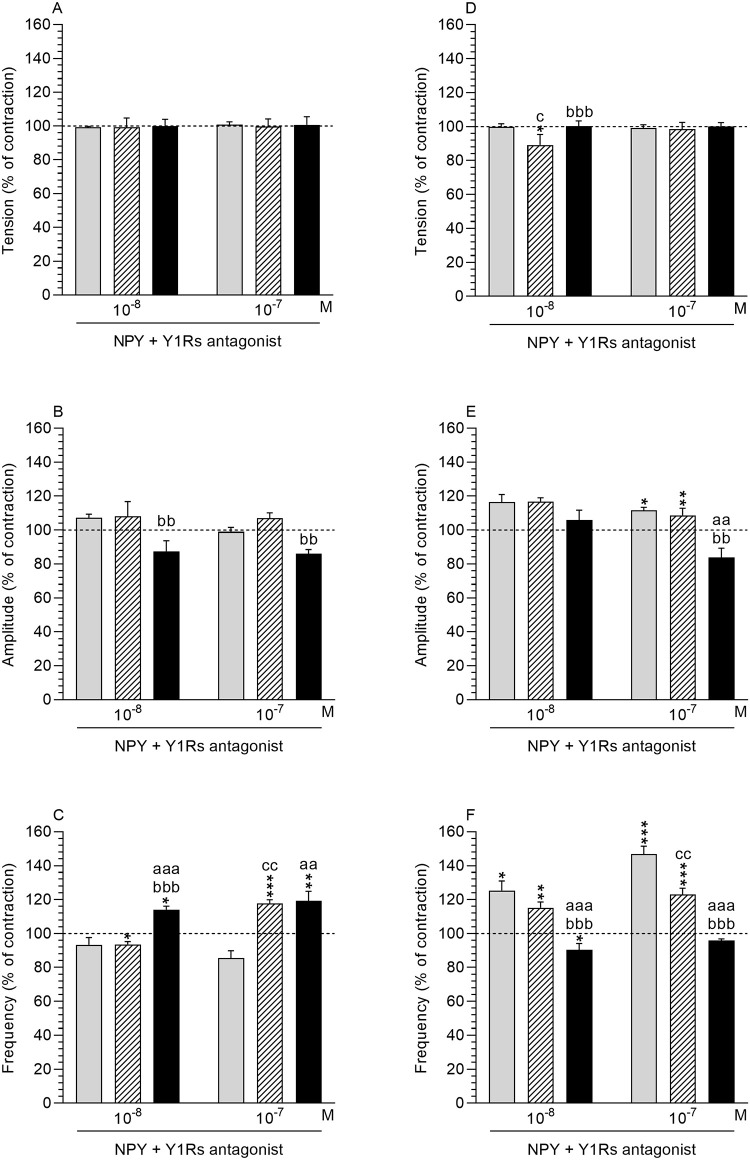
Effect of NPY in the presence of Y1Rs antagonist. Effect of neuropeptide Y (NPY) on the tension (A, B), amplitude (C, D) and frequency (E, F) of contraction in myometrium (A-C) and endometrium/myometrium (D-F) strips from the control (grey bars), saline- (hatched bars) and *E*. *coli* (black bars) -treated uteri of gilts in the presence of Y1Rs antagonist. Data obtained from five experiments (gilts, in each group). Effects of Y1Rs antagonist and particular doses of NPY are presented as a percentage (mean±SEM) in relation to the basal (pre-treatment period) tension, amplitude and frequency, accepted as 100% (horizontal lines). *P<0.05, **P<0.01, ***P<0.001—indicate differences in comparison to the basal value in each group; ^aa^P<0.01, ^aaa^P<0.001—indicate differences between the control and *E*. *coli*-treated groups for the same treatment; ^bb^P<0.01, ^bbb^P<0.001- indicate differences between saline- and *E*. *coli*-treated groups for the same treatment; ^c^P<0.05, ^cc^P<0.01—indicate differences between the control and saline-treated groups for the same treatment.

*NPY effect in comparison to the period before antagonist and NPY treatment*. *Tension and amplitude*: Following the use of Y1Rs antagonist and NPY (10^−8^, 10^−7^ M), the tension and amplitude in the MYO of all studied groups did not significantly change (Fig 6A and 6B).

*Frequency*: In response to Y1Rs antagonist and NPY at a dose of 10^−8^ M in the MYO of the saline-treated uteri, a drop (P<0.05) in the frequency was noted, while it increased (P<0.05) in the *E*. *coli*-treated uteri (Fig 6C). A rise in the value of this parameter was also found in the MYO of the saline- (P<0.001) and bacteria (P<0.01) -treated uteri in response to Y1Rs antagonist and NPY at a dose of 10^−7^ M.

*Comparison of antagonist and NPY effect between groups*. *Tension*: Following the use of Y1Rs antagonist and NPY (10^−8^, 10^−7^ M) the tension in the MYO did not differ significantly between the control, saline- and *E*. *coli*-treated gilts (Fig 6A).

*Amplitude*: After application of Y1Rs antagonist and NPY (10^−8^ and 10^−7^ M) the amplitude in the MYO of the inflamed uteri was lower (P<0.01) than in the saline-injected uteri (Fig 6B). *Frequency*: In response to Y1Rs antagonist and NPY at a dose of 10^−8^ M the frequency in the MYO of the inflamed uteri was higher (P<0.001) compared to the control and saline-treated uteri (Fig 6C). In the MYO of the saline and *E*. *coli*-treated uteri, the values of this parameter were higher (P<0.01) in response to the Y1Rs antagonist and NPY at a dose of 10^−7^ M than in the control uteri.

#### Endometrium/myometrium

*NPY effect in comparison to the period before antagonist and NPY treatment*. *Tension*: Following the use of Y1Rs antagonist and NPY at a dose of 10^−8^ M, the tension in the ENDO/MYO of the saline-treated uteri was decreased (P<0.05) (Fig 6D).

*Amplitude*: In the presence of Y1Rs antagonist, NPY at a dose of 10^−7^ M increased the amplitude in the ENDO/MYO of the control (P<0.05) and saline-treated (P<0.01) uteri (Fig 6E).

*Frequency*: Following the use of Y1Rs antagonist together with NPY at a dose of 10^−8^ M, the frequency increased in the ENDO/MYO of the control (P<0.05) and saline (P<0.01) -treated uteri, while it decreased in the *E*. *coli*-treated uteri (P<0.05) (Fig 6F). A rise (P<0.001) in the frequency was also noted in response to Y1Rs antagonist and NPY at a dose of 10^−7^ M in ENDO/MYO of the control and saline-treated uteri.

*Comparison of antagonist and NPY effect between groups*. *Tension*: Following the use of Y1Rs antagonist and NPY at a dose of 10^−8^ M, the tension in the ENDO/MYO of the saline-injected uteri was lower than in the control (P<0.05) and *E*. *coli*-treated (P<0.001) uteri (Fig 6D).

*Amplitude*: In the presence of Y1Rs antagonist and NPY at a dose of 10^−7^ M, the amplitude in the ENDO/MYO of the inflamed uteri was lower (P<0.01) than in the control and saline-injected uteri (Fig 6E).

*Frequency*: Following the use of this antagonist and NPY at doses of 10^−8^ and 10^−7^ M, the frequency in the ENDO/MYO of the inflamed uteri was reduced (P<0.001) compared to the control and saline-treated uteri (Fig 6F). In response to Y1Rs antagonist and NPY at a dose of 10^−7^ M, the frequency in the ENDO/MYO of the saline-treated uteri was lower (P<0.01) than in the control uteri.

### Effect of NPY in the presence of Y2Rs antagonist

#### Myometrium

**Fig 7:**
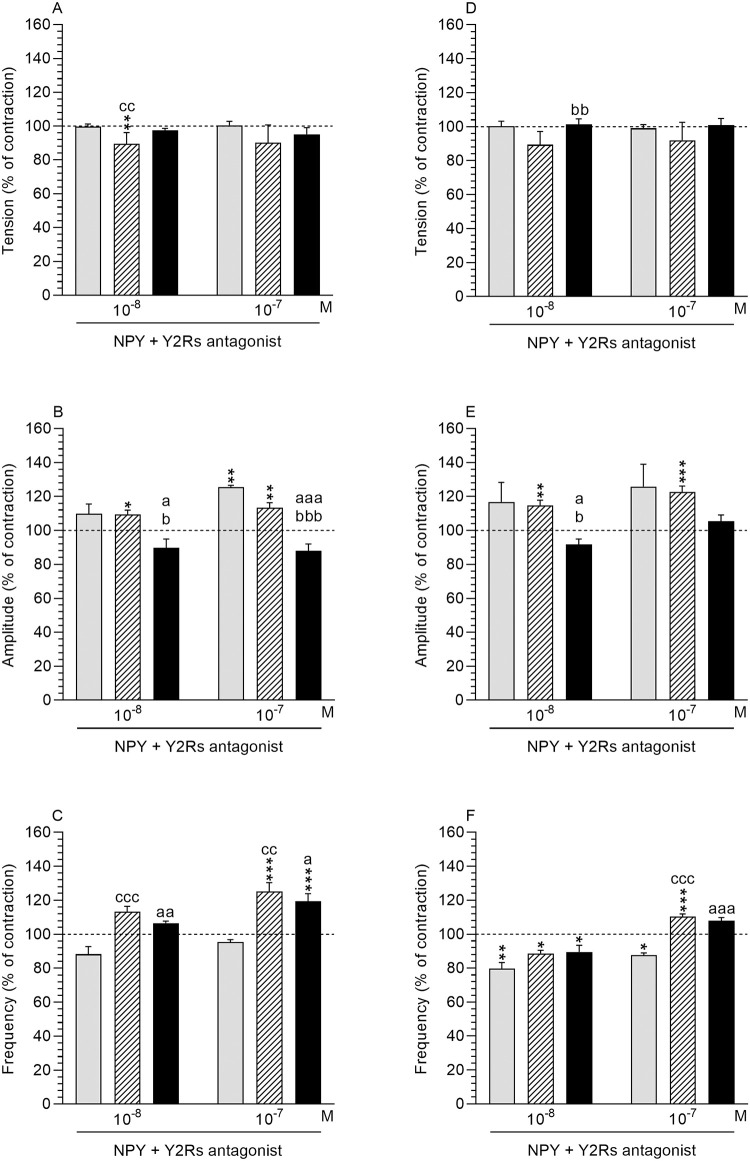
Effect of NPY in the presence of Y2Rs antagonist. Effect of neuropeptide Y (NPY) on the tension (A, B), amplitude (C, D) and frequency (E, F) of contraction in myometrium (A-C) and endometrium/myometrium (D-F) strips from the control (grey bars), saline- (hatched bars) and *E*. *coli* (black bars) -treated uteri of gilts in the presence of Y2Rs antagonist. Data obtained from five experiments (gilts, in each group). Effects of Y2Rs antagonist and particular doses of NPY are presented as a percentage (mean±SEM) in relation to the basal (pre-treatment period) tension, amplitude and frequency, accepted as 100% (horizontal lines). *P<0.05, **P<0.01, ***P<0.001—indicate differences in comparison to the basal value in each group; ^a^P<0.05, ^aa^P<0.01, ^aaa^P<0.001—indicate differences between the control and *E*. *coli*-treated groups for the same treatment; ^b^P<0.05, ^bb^P<0.01, ^bbb^P<0.001- indicate differences between saline- and *E*. *coli*-treated groups for the same treatment; ^cc^P<0.001, ^ccc^P<0.001- indicate differences between the control and saline-treated groups for the same treatment.

*NPY effect in comparison to the period before antagonist and NPY treatment*. *Tension*: The tension in the MYO of the saline-treated uteri was lowered (P<0.01) in response to Y2Rs antagonist and NPY at a dose of 10^−8^ M (Fig 7A).

*Amplitude*: The amplitude in the MYO of the saline-treated uteri was increased (P<0.05) after use of Y2Rs antagonist and NPY at a dose of 10^−8^ M (Fig 7B). A similar situation (P<0.01) was revealed in the MYO of the control and saline-treated uteri in response to Y2Rs antagonist and NPY at a dose of 10^−7^ M.

*Frequency*: After treatment with Y2Rs antagonist and NPY at a dose of 10^−7^ M the frequency in the MYO of the saline- and *E*. *coli*-treated uteri was increased (P<0.001) (Fig 7C).

*Comparison of antagonist and NPY effect between groups*. *Tension*: In the saline-treated uteri, after the use of Y2Rs antagonist and NPY at a dose of 10^−8^ M, the tension in the MYO was lower (P<0.01) than in the control uteri (Fig 7A).

*Amplitude*: The amplitude in the MYO of the inflamed uteri was decreased in response to Y2Rs antagonist and NPY at doses of 10^−8^ (P<0.05) and 10^−7^ (P<0.001) M as compared to the control and saline-treated uteri (Fig 7B).

*Frequency*: In the MYO of *E*. *coli*-injected uteri, the use of Y2Rs antagonist and NPY at doses of 10^−8^ (P<0.01) and 10^−7^ (P<0.05) M led to an increase in the frequency as compared to the control uteri (Fig 7C). The frequency in the MYO of the saline-treated uteri was higher after the application of Y2Rs antagonist and NPY at doses of 10^−7^ (P<0.001) and 10^−8^ (P<0.01) M than in the control uteri.

#### Endometrium/myometrium

*NPY effect in comparison to the period before antagonist and NPY treatment*. *Tension*: The tension in ENDO/MYO of the control, saline- and *E*. *coli*-injected uteri did not significantly change in response to Y2Rs antagonist and NPY (10^−8^, 10^−7^ M) (Fig 7D).

*Amplitude*: The amplitude rose in the ENDO/MYO of the saline-treated uteri in response to Y2Rs antagonist and NPY at doses of 10^−8^ (P<0.01) and 10^−7^ M (P<0.001) (Fig 7E).

*Frequency*: After treatment with Y2Rs antagonist and NPY at a dose of 10^−8^ M, the frequency in the ENDO/MYO of the control (P<0.01) and saline- and *E*. *coli* (P<0.05) -treated uteri was decreased (Fig 7F). A similar effect (P<0.05) was also exerted by this antagonist and NPY at a dose of 10^−7^ M in the ENDO/MYO of the control uteri. After treatment with Y2Rs antagonist and NPY at a dose of 10^−7^ M, the frequency in the ENDO/MYO of the saline-treated uteri was increased (P<0.001).

*Comparison of antagonist and NPY effect between groups*. *Tension*: In the ENDO/MYO of the *E*. *coli*-injected uteri, after the use of Y2Rs antagonist and NPY at a dose of 10^−8^ M the tension was higher (p<0.01) than in the saline-treated uteri (Fig 7D).

*Amplitude*: The amplitude in the ENDO/MYO of the inflamed uteri was decreased (P<0.05) in response to Y2Rs antagonist and NPY at a dose of 10^−8^ M compared to the control and saline-treated uteri (Fig 7E).

*Frequency*: After using Y2Rs antagonist and NPY at a dose of 10^−7^, the frequency in the ENDO/MYO of the control uteri was lower (P<0.001) than in the saline- and *E*. *coli*-treated uteri (Fig 7F).

## Discussion

This report shows the expression patterns of Y1Rs and Y2Rs in the gilt inflamed uteri and the role of NPY and these receptors in the contractile activity of pathologically-changed organs. The results of macroscopic and histopathological examination of uteri utilized in the current experiment were presented previously. In the *E*. *coli-*injected uteri, severe acute endometritis was revealed. In the ENDO, edema and hyperemia, the increased number of neutrophils and the damage of luminal and/or glandular epithelium were revealed [33].

To date, in functional studies concerning the characterization of myometrial contractile response to NPY under physiological conditions, only the role of Y1Rs in the rat MYO was indicated [19]. The present report, for the first time, shows Y1R and Y2R expression in the myometrial tissues. Significant differences in the Y1R and Y2R mRNA and protein expression between the CON and SAL groups were not revealed, which indicates that the administration of saline into uterus did not affect these receptor levels.

A completely new indication of the current research is that inflammatory state changes the Y1R and Y2R expression in the MYO. Intrauterine *E*. *coli* injection led to a drop in the levels of Y1R mRNA and protein expression and to a rise in Y2R protein expression, compared to the CON and SAL groups. The elevated protein levels of Y2Rs and the lack of changes in the mRNA expression of Y1Rs and Y2Rs in the heart tissue of diabetic patients were presented [39]. In turn, the expression of mRNA Y1Rs was increased in uterosacral ligaments in women with pelvic organ prolapse [28]. In the present study, only the changes in Y1Rs mRNA expression in the MYO after intrauterine injection of *E*. *coli* in relation to the CON and SAL groups were coincident with the product of this gene. In the MYO of *E*. *coli* group, the unchanged mRNA level of Y2Rs was accompanied by a rise in this receptor protein level.

Immunofluorescent analysis (present study) revealed that inflammation did not affect the distribution Y1Rs and Y2Rs in the MYO, i.e. both receptors were found in the muscle and blood vessel cells in gilts of the *E*. *coli*, SAL and CON groups. This suggests that under physiological and inflammatory conditions these myometrial cells are target cells for NPY.

The changes in the expression of Y1Rs mRNA and protein and Y2Rs protein found in the current study could result from bacterial infection and the associated inflammation of ENDO. The alterations in Y1Rs and Y2Rs expression may have been caused by lipopolysaccharide released from bacteria [3] and inflammatory cytokines synthetized in large amounts [40, 41] in the infected uterus, although this particular issue needs further study. The Y1Rs and Y2Rs expression may also be modulated by the steroid hormones. Earlier, both the ability of testosterone to modulate of Y1Rs expression in vascular smooth muscle cells in rat testes [42] and of estrogen to stimulate this receptor expression in human breast cancer cell line [43] were presented. It is important that in the gilts suffering from endometritis, peripheral blood levels of estrogens and progesterone were decreased, androstenedione level was increased [44].

Considering the changes in the levels of Y1R and Y2R expression in the MYO of the inflamed uteri, and the immunoreaction for these receptors in myometrial structures, it is suggested that Y1Rs and Y2Rs are involved in the function of the MYO of the uteri with inflammation. The presence of Y1Rs and Y2Rs in the muscle layer of blood vessels of the *E*. *coli* group (also the CON and SAL groups) probably explains the involvement of these receptors in the regulation of myometrial blood flow by NPY. Earlier, it was shown that NPY action on the contractile activity of guinea pig uterine arteries [23, 24] is mediated via Y1Rs [25] and of rabbit ovarian artery via Y1Rs and Y2Rs [15].

As it was mentioned above, the impairment of uterine contractility plays an important role in the generation and regulation of inflammation [3, 4]. In reference to this, the present study also focused on the effect of NPY on the contraction of inflamed uteri. The NA influence on the contractile activity of uteri utilized in the current experiment, confirming their viability and usefulness for study, was reported earlier. This revealed that NA dropped the contractility of uteri of gilts from the CON, SAL and *E*. *coli* groups [32].

It was found that NPY did not change the tension, but either decreased or had no effect on the amplitude and frequency in the CON and SAL groups, compared to the period before NPY treatment. These findings are in contrast to that described in rabbit MYO [18] and convergent to that reported in the colon [29]. In the rat MYO, short-term NPY stimulation decreased contractile response to NPY, while long-term NPY infusion increased it [19]. NPY did not change motility in the rat [20] and human [22] uteri. The current study shows that response of the same tissue to NPY at the same dose differed partly in the CON and SAL groups compared to the period before NPY treatment. However, as revealed in this study, in the MYO of both groups, the levels of Y1R and Y2R mRNA and protein expression were similar. It may be the case that the partial differences between reaction of the CON and SAL groups to NPY may result from MYO distribution of Y1Rs and Y2Rs and their sensitivity changed after intrauterine saline injection rather than Y1Rs and Y2Rs expression.

This study for the first time presents the NPY role in the contractility of the inflammatory-changed uterus. As compared to the period before NPY application, in the *E*. *coli* group, as in the CON and SAL groups, this neuropeptide did not alter the tension, decreased or not affect amplitude and frequency, However, in the *E*. *coli* group, the frequency changed more often than the amplitude, while in the CON and SAL groups the response was reversed. In the inflamed uteri, the amplitude was higher than in the control and/or saline-treated uteri, and the frequency was lower than in the saline-treated uteri.

In the present study the partly different values of the amplitude and frequency were also revealed between the MYO and ENDO/MYO of gilts from the CON, SAL and *E*. *coli* groups in response to the same doses of NPY. These parameters were changed more often in the MYO than in the ENDO/MYO. This may be a consequence of the modulating effect of NPY on other neurotransmitters. It is known that NPY regulates the NA [22, 26] and VIP [18, 23] influence on uterine blood flow. NPY also inhibits the contractility mediated by cholinergic nerve fibers in the rat uterine cervix [20].

Furthermore, this study shows that the use of NPY together with Y1Rs and Y2Rs antagonists caused changes in the values of amplitude and frequency in the CON, SAL and *E*. *coli* groups, and the tension in SAL group, as compared to the NPY effect alone. It was found that in the CON and SAL groups, NPY reduced the contractile activity of the uterus by both Y1Rs and Y2Rs. To date, only studies concerning the functional role of YRs in uterine contractility under physiological conditions and the participation of Y1Rs in NPY action have been presented [19]. Thus, the current report is the first in which the role of Y2Rs in uterine contractility was shown.

As it was mentioned above, the importance of YRs in NPY affected contractility of the inflamed uterus was not investigated. This study demonstrated that NPY reduces the contractile activity of the inflammatory-changed uterus by Y1Rs and Y2Rs. However, it should be emphasized that in the inflamed uteri, NPY lowered the amplitude more weakly and the frequency more strongly compared to healthy uteri. Taking into consideration the expression of YRs examined in the current study, it is assumed that this NPY effect on amplitude in the inflamed uteri may result from lowered Y1R mRNA and protein expression in the MYO. Moreover, despite the higher content of Y2Rs protein, their stimulation by NPY could be insufficient to further lower the amplitude in the inflamed uterus. The enhanced myometrial expression of Y2Rs may lead to a greater frequency reduction. It is worth noting that the NPY action on contractility on the inflamed uteri may also result from the different MYO distribution of particular subtypes of YRs and their different ligand affinities. As it was noted above, NPY has the ability to inhibit contractility mediated by cholinergic nerve fibers in the rat uterine cervix [20] and in the rabbit urethra [45]. This suggests that NPY could decrease the contractility of inflamed (also healthy) uteri, also indirectly by modulating release and the action of other neurotransmitters, for example, NA and ACh. These neurotransmitters, as shown in earlier studies, decrease the contractility of the inflamed porcine uterus [32]. However, this supposition requires research. The effect of NPY on the contractile activity of the porcine inflamed uterus found in the present study suggests that NPY, besides NA and ACh [32], as well as metabolites of arachidonic acid [6, 8, 9, 10], may play an essential role in the function of the pathologically-changed organ. It should, however, be emphasized that the relaxing effect of NPY (a weaker action on amplitude and stronger action on frequency) may be important for the collection of inflammatory exudate in the uterine cavity. This can lead to prolonged inflammation and, as a result, to fertility problems and economic losses.

## Conclusions

This study demonstrated that inflammation changes the expression of Y1Rs and Y2Rs in the MYO of gilts and that NPY decreases the contractile activity of the porcine inflamed uterus, acting by Y1Rs and Y2Rs. Thus, reduced uterine contractile activity during spontaneous inflammation may be NPY dependent. However, the mechanisms underlying changes in the YRs expression and their role in an inflamed uterus should be further explored. The use of pharmacological modulators of Y1R and Y2R may contribute to an increase in the contractile activity of the inflamed uterus.
